# Mechanical ventilation weaning issues can be counted on the fingers of just one hand: part 2

**DOI:** 10.1186/s13089-020-00160-z

**Published:** 2020-03-13

**Authors:** Luigi Vetrugno, Alessandro Brussa, Giovanni Maria Guadagnin, Daniele Orso, Francesco De Lorenzo, Gianmaria Cammarota, Erminio Santangelo, Tiziana Bove

**Affiliations:** 1 Anesthesiology and Intensive Care Clinic, Department of Medicine, University of Udine, Via Colugna 50, 33100 Udine, Italy; 2 Department of Anesthesiology and Intensive Care, Maggiore della Carità University Hospital, Corso Mazzini 18, 28100 Novara, Italy; 3 Department of Translational Medicine, University of Eastern Piemonte, via Solaroli, 17, 28100 Novara, Italy

**Keywords:** Weaning, Mechanical ventilation, Lung ultrasound, Diaphragm dysfunction, Neurally adjusted ventilation assist

## Abstract

Assessing heart and diaphragm function constitutes only one of the steps to consider along the weaning path. In this second part of the review, we will deal with the more systematic evaluation of the pulmonary parenchyma—often implicated in the genesis of respiratory failure. We will also consider the other possible causes of weaning failure that lie beyond the cardio-pulmonary-diaphragmatic system. Finally, we will take a moment to consider the remaining unsolved problems arising from mechanical ventilation and describe the so-called protective approach to parenchyma and diaphragm ventilation.

## Lung ultrasound score (LUS)

**Fig. 1:**
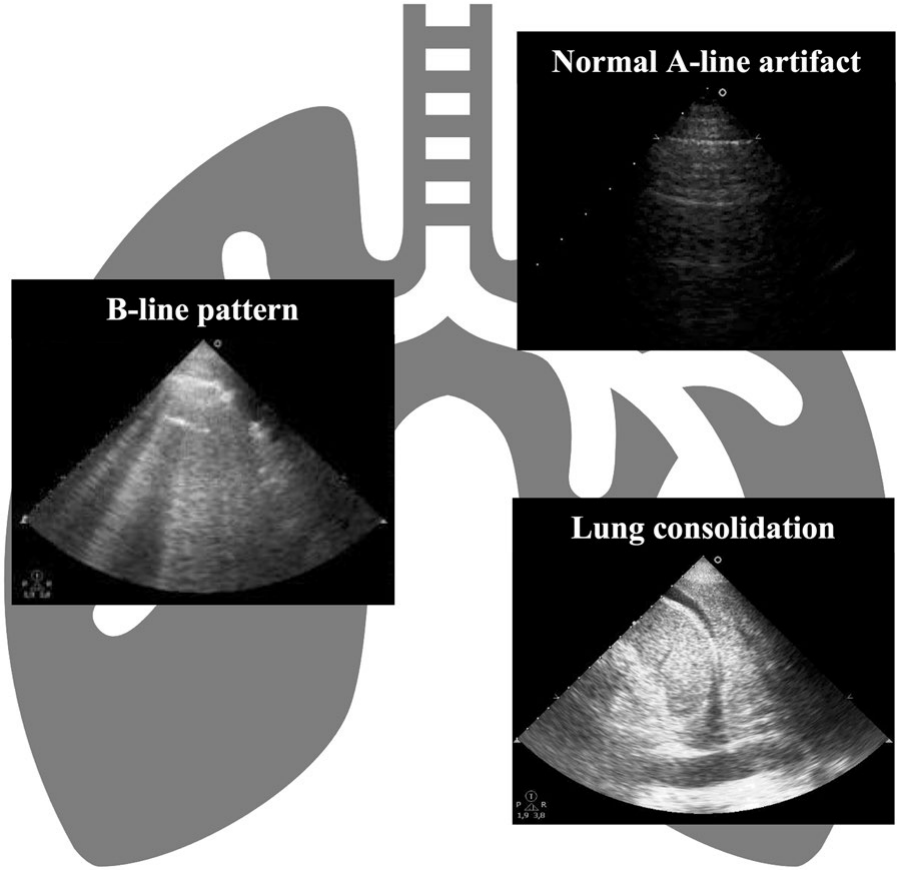
Different echographic patterns during lung ultrasound evaluation

The lung ultrasound (LUS) score is a useful tool that quantifies aerated lung mass and provides real-time information during mechanical ventilation and weaning (Fig. 1) [1]. Specifically, its application in the clinical setting enables optimization of the ventilatory settings in challenging patients by estimating the recruitability of poorly aerated pulmonary mass. This allows clinicians to monitor lung aeration during the weaning process, providing a positive prognostic factor. The LUS score may also help discriminate between a cardiac, parenchymal, or diaphragmatic cause of loss of lung aeration [2] and, in turn, help identify the most appropriate therapeutic approach.

Bouhemad and colleagues (2010) were the first to postulate the potential usefulness of LUS as a tool for monitoring parenchymal reaeration. Their studies involved assigning a positive or negative score to reflect the change occurring in the parenchymal ultrasound pattern as a result of adjustments to medical antibiotic therapy [3] or positive end-expiratory pressure (PEEP) titration [4]. They found that the degree of lung reaeration could be accurately estimated using the LUS reaeration score. However, use of the LUS score to set ventilatory parameters resulted in the inability to predict lung over-inflation—the physiopathological basis for ventilation-induced lung injury. Soon after, Stefanidis et al. reached the same conclusions in a small pilot study, which once again pointed out the reliability of LUS in evaluating parenchymal reaeration in acute respiratory distress syndrome (ARDS) patients during a PEEP trial [5]. In a more recent, prospective, multicenter study, Haddam et al. analyzed changes in LUS score during the prone positioning of patients with severe ARDS. They revealed that even though the LUS score provides a way to consistently monitor changes in regional lung aeration, it does not correlate with improvements in blood gas oxygenation. A number of reasons may explain this, including a short prone-positioning trial before blood gas analysis and a less permissive cut-off in evaluating PaO_2_ improvements. Nevertheless, Haddam and colleagues underlined the feasibility and utility of LUS evaluation, practically considering it a routine technique [6]. The capacity of LUS to evaluate lung reaeration was also confirmed by Chinardet et al. who used it to estimate lung reaeration following pleural drainage in ARDS patients [7]. However, a recent randomized crossover study by Chiumello et al. found that variations in the LUS score did not correlate with PEEP-induced lung recruitment due to a still inaccurate definition of consolidation pattern [8]. These results could open up a new line of research aimed at increasing the specificity of ultrasound evaluation.

The monitoring of lung aeration during the weaning process embodies another essential aspect of LUS evaluation since it indirectly leads to successful extubation [9]. Clear evidence of the usefulness of the LUS score in the weaning process is provided in the metanalysis by Llamas-Álvarez et al. in which ultrasound evaluation is concluded to provide valuable information in predicting weaning outcome [10]. Tenza-Lozano et al. also came to this conclusion and went on to propose a new and more reproducible score that only considers the anterior, lateral, and posterior-basal thoracic areas [11]. Soummer et al. showed that an LUS score < 13 after a spontaneous breathing trial (SBT) correlates with successful weaning and safe extubation, whereas a LUS score > 17 predicts weaning failure with post-extubation respiratory distress [12]. The association between lung de-recruitment and weaning failure was emphasized by Jabaudon et al. who found a loss of aerated lung mass to be the only predictor of weaning failure [13]. In addition to corroborating all of the above, Haji et al. also pointed out the clinical relevance of LUS examination in predicting weaning outcome [14].

The role of LUS as a prognostic factor was first suggested by Frassi et al. [15], who showed that the number of B-lines in an ultrasound evaluation correlated with mortality rate in a cardiac subgroup of patients admitted to hospital with dyspnea or chest pain. Extending this concept, Zhao et al. [16] demonstrated how LUS B-line number could be useful in accurately assessing extravascular lung water in ARDS patients. LUS score also correlates with the lung injury score, the PaO_2_/FiO_2_ ratio, static compliance, and, above all, mortality risk, providing a new and reliable prognostic tool for ARDS patients. More recently, Zou et al. [17] broadened the prognostic value of LUS score to include shock intensive care unit (ICU) patients, and showed that a modified LUS score may act as an independent risk factor for ICU mortality. These data on shock patients were confirmed by Yin et al. [18] in a study involving 175 patients in which the LUS score correlated with 28-day mortality as well as the APACHE II score and lactate serum levels.

Lung ultrasonography, together with its growing role as a prognostic factor, has even been applied in the differential diagnosis of respiratory failure etiology [19]. Jambrik et al. [20] performed the first assessment of extravascular lung water using lung sonography and from these first data LUS has progressed from being the primarily technique to quantify pulmonary interstitial edema, which may derive from different causes, to being developed into a new diagnostic tool for identifying the origin of aerated lung loss. Indeed, over the years, the breadth of application of LUS has expanded, and it is now being applied in the assessment of extravascular lung water in hemodialysis patients [21] and in heart failure patients [22], not to mention the already cited role of the LUS score in predicting a successful SBT [12, 23].

Considering all of the above evidence, the critical role of lung ultrasonography and its various applications in the care of critically ill patients becomes highly evident. The role of the anesthesiologist in the management of this powerful tool is thus paramount in order to provide patients with the best medical treatment.

## Out of the box

### Neurocritical patients

Mechanical ventilation is often necessary in brain injury patients (i.e., those suffering from ischemic or hemorrhagic stroke, or severe brain trauma, or metabolic brain injury or resulting from alcohol intoxication, and neurosurgery patients) to prevent aspiration, hypoxemia, hypercapnia and further brain injury resulting from reduced/abolished airway protective mechanisms and respiratory drive. Unfortunately, data about mechanical ventilation settings and the weaning process in neurocritical patients are lacking in the literature. A minimal level of arousal should be obtained before weaning and extubation, but neurocritical patients frequently experience prolonged mechanical ventilation periods because of our inability to understand the patient’s state of consciousness [24]. The minimal level of arousal necessary to achieve successful extubation also remains controversial. The Glasgow Coma Scale (GCS) has never been validated in intubated patients and, when employed, it has proved to be an inconsistent predictor of successful extubation if used alone [25, 26]. Several scores have been proposed to assess patient arousal, combining airway and neurological statuses, but all lack external validation and often they are not easily applicable at the bedside. In cases of prolonged diminished levels of consciousness, neurocritical patients may never undergo the weaning process, and tracheostomy is commonly employed in the management of these patients, but the timing of this procedure is controversial. Prolonged invasive mechanical ventilation (IMV) in this category of patients can lead to higher rates of ventilator-acquired pneumonia, increased ICU length of stay, and higher mortality rates [24, 25, 27] compared with non-neurological critically ill patients [28].

### Intra-abdominal hypertension (IAH)

Intra-abdominal hypertension (IAH) is defined as an abdominal pressure that exceeds 12 mmHg in at least three consecutive assessments performed within a time window of 4–6 h. Abdominal hypertension can transform into abdominal compartment syndrome (ACS) when intra-abdominal pressure (IAP) reaches values above 20 mmHg with associated single or multiple organ dysfunction/failure [29].

The prevalence of IAH varies in the literature according to the different patient populations studied; it stands at around 54% in medical ICU patients, 65% in surgical ICU patients, and 32% in combined medical–surgical ICU patients [30, 31]. IAH is a serious problem that can produce adverse effects involving both abdominal (i.e., bowel, kidney) and extra-abdominal (i.e., cardiovascular, respiratory) organs, and it is recognized as an independent indicator of mortality in critically ill patients [32].

**Fig. 2:**
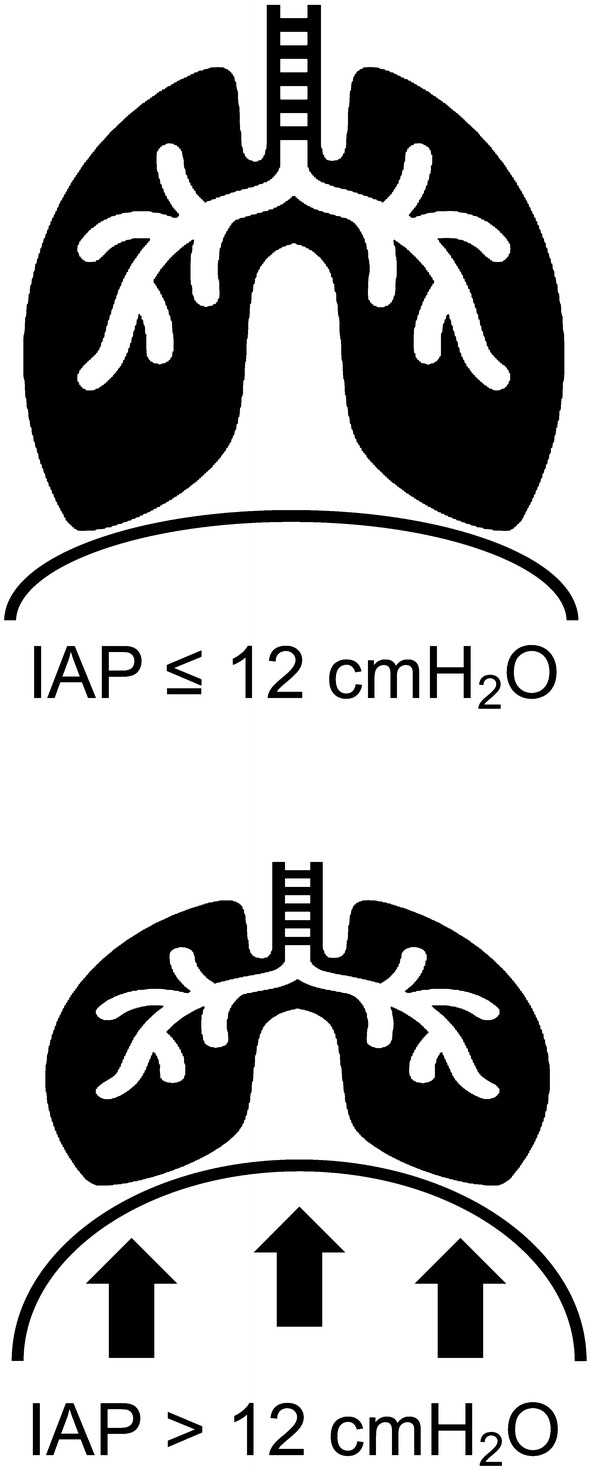
Effect of intra-abdominal pressure on diaphragmatic dome. *IAP* intra-abdominal pressure

Primary causes of IAH (i.e., pneumoperitoneum, hemoperitoneum, abdominal trauma, pancreatitis, liver transplantation) can lead to ARDS, and primary ARDS may develop into IAH. The primary mechanical effects through which IAH affects respiratory function are a decreased compliance of the thoracic–pulmonary system and an upward displacement of the diaphragm’s starting position [33]. As the lower inflection point of the pressure–volume curve shifts to the right, alveolar opening pressure and inspiratory pressures are also increased [33]. All these elements cause an increased respiratory workload for the spontaneously breathing patient, a compensatory increase in frequency, and, if prolonged, muscle exhaustion (Fig. 2). As a result of reduced inspiratory flow and compression of the lung parenchyma, the dependent areas of the lung will tend toward atelectasis [33]. Subsequently, the continued presence of IAH generates secondary acute lung injury [33], i.e., barotrauma, linked to the increase in peak and plateau pressure in the respiratory tract with the activation of a neutrophilic immune response [33]. Atelectasis may trigger the development of pneumonia [33].

Despite some disputes in literature about the effects of mechanical ventilation and PEEP on IAP, ARDS combined with mechanical ventilation is a known risk factor for intra-abdominal hypertension. The use of PEEP increases alveolar recruitment, in turn, increasing residual functional capacity and oxygenation [34], although it causes a flattening of the diaphragmatic dome and a shift to the right side of the pressure–volume curve with the applied pressure being subsequently directed to the abdominal cavity [35]. As such, PEEP should be employed with caution in ARDS patients with IAH, also because moderate PEEP levels can increase IAP and significantly decrease abdominal perfusion pressure proportionally to the PEEP applied [36].

Given the interdependence of the thoracic and abdominal cavities, it is unsurprising that weaning from mechanical ventilation can be particularly difficult in the presence of IAH [37]. We suggest, therefore, in agreement with various other publications and international recommendations, that IAP is closely monitored in mechanically ventilated patients with ARDS.

### Pleural effusion

**Fig. 3:**
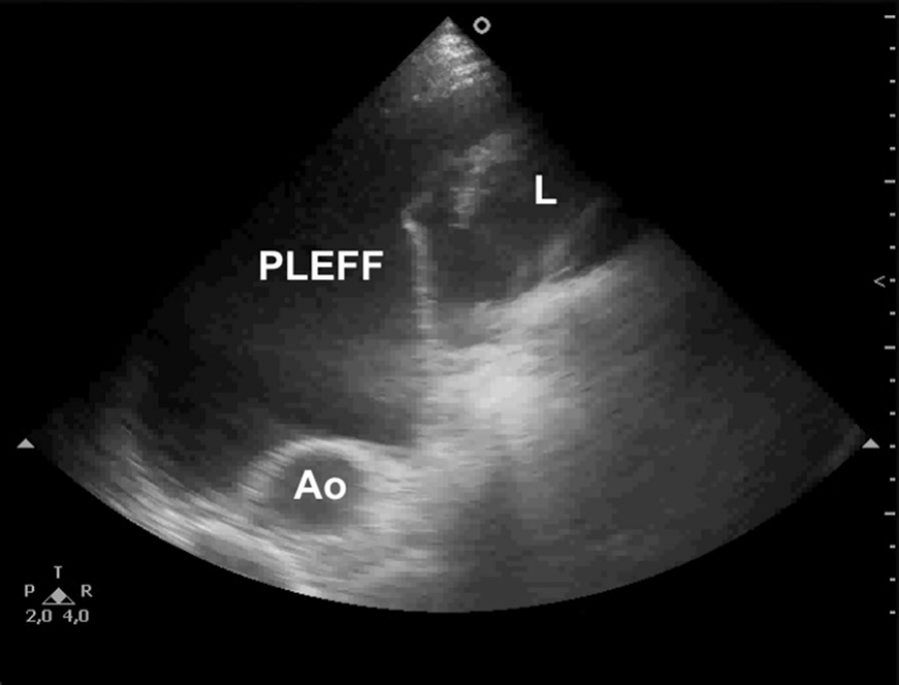
Massive left-sided pleural effusion with floating fibrin deposition. *PLEFF* pleural effusion, *Ao* aorta, *L* lung

Pleural effusion (PLEFF) is common in the ICU, although the incidence varies according to the diagnostic technique being used (8% to 60%) [38]. However, its role in weaning failure has never been demonstrated—even though the amount of PLEFF is known to influence diaphragm force [39], but this has never been considered in the context of weaning. PLEFF can exacerbate gas exchange, respiratory dynamics, and hemodynamic stability, whereas pleural drainage can improve oxygenation and respiratory mechanics (Fig. 3). At the time of this review, doubts persist about the effectiveness of pleural drainage. In a previous publication, we addressed the effectiveness of ultrasound-guided placement of a small-bore pleural drain for improving patient respiratory gas exchange in terms of the PaO_2_/FiO_2_ ratio, but no correlation with the maintenance of spontaneous breathing or with weaning from mechanical ventilation was found, in agreement with the work by Dres et al. [40]. These results support the concept that PLEFF cannot constitute the sole parameter being considered when weaning a patient from the ventilator. However, the inconsistency in PLEFF estimations obtained using ultrasound necessitates the need to standardize the methods used for assessing pleural effusion volume (an issue that is overlooked in the current international recommendations on lung ultrasound) [41, 42]. That said, PLEFF drainage also provides the opportunity to obtain chemical–physical and cytological samples to guide the differential diagnosis of PLEFF and any follow-up therapy.

### Mechanical ventilation: unsolved issues and possible solutions

Mechanical ventilation itself may cause lung, heart, and diaphragm dysfunction (Table 1). The mechanisms underlying these types of alterations are not entirely known. The concept of lung and heart protection during mechanical ventilation is better established, whereas a significant gap exists in the literature regarding diaphragm protection. In fact, while specific protocols of lung-protective ventilation are available, the diaphragm is very often neglected, despite the fact that it constitutes the primary actor in physiological respiration.

**Table 1:** Benefits and risks deriving from invasive mechanical ventilation

Benefits	Risks
Organ support during acute respiratory illness	VILI
	VAP
Protective ventilation protocols	VIDD
	Overassistance
	Oversedation

*VILI* ventilation-induced lung injury, *VAP* ventilator-acquired pneumonia, *VIDD* ventilator-induced diaphragm dysfunction

#### Ventilator-induced lung injury (VILI)

The literature is rich with evidence and explanations regarding how mechanical ventilation can cause lung injury. Excessive pressure (barotrauma) or volume (volutrauma) and cyclic alveolar opening–closing (atelectotrauma) are the components that cause ventilator-induced lung injury (VILI) [43]. The effects of all three mechanisms occur under the broader term of “biotrauma”, which defines the inflammatory process that follows the initial injury [44]. Specifically, ventilation pressure and induced tidal volume are closely interconnected in the genesis of parenchymal damage. Barotrauma is due to excessive stress applied to the lung, directly proportional to the transpulmonary pressure, while volutrauma is secondary to an excessive parenchymal strain, correlated to the tidal volume [45]. In other words, harmful levels of pressure and volume are the two sides of the same coin. Atelectotrauma refers to the continuous opening and closure of potentially recruitable alveoli, which, mainly due to the poor distribution of the pulmonary surfactant, tend to open in response to increasing pressures during the inspiratory phase and to close in the expiratory phase, in accordance with Laplace’s law [46]. In the long run, this mechanism triggers the inflammatory process and progressive alveolar atelectasis.

More recent observations regarding IMV have also considered respiratory flow and frequency among the possible determinants of lung injury. As for the aforementioned relationship between volume and pressure, once again, lung injury can derive from the interdependence between inspiratory flow and respiratory rate. To explain this complicated process in more simple terms: the inspiratory flow conveys a given tidal volume based on the pressure delivered in a unit of time, which is, in turn, determined by the respiratory rate and the I:E ratio (inspiration:expiration ratio). The higher the respiratory rate, the lower the inspiratory time and the higher the flow, resulting in greater pressure applied to the lung parenchyma and an increased risk of VILI [47].

From the fifth day of IMV onward, the risk of ventilator-associated pneumonia increases and its presence is correlated with increased morbidity and mortality [48].

#### Ventilator-induced diaphragm dysfunction

Mechanical ventilation may also lead to a “…loss of diaphragmatic force-generating capacity” [49], causing ventilator-induced diaphragm dysfunction (VIDD) [49]—an underdiagnosed condition in clinical practice. The causes of this type of diaphragmatic weakness may be prolonged inactivity during mandatory or assisted mechanical ventilation (underloading, i.e., due to the use of myorelaxants or excessive positive pressure support) or an excessive workload (overloading; i.e., due to ventilator asynchrony or inadequate pressure support) [50–53]. Diaphragmatic weakness in critically ill patients in the ICU may also form part of a more general picture of acquired myopathy or polyneuropathy associated with critical illness, or it may present as a single dysfunction in patients who otherwise present no signs of multiple district involvement [54]. Histological changes (oxidative stress and proteolysis phenomena) characterize the pathophysiology of VIDD, which ultimately lead to muscle atrophy [55].

#### Searching for more protective ventilation

The demonstration that intrinsic damage resulted from mechanical ventilation led to the development of ventilation protocols able to prevent or at least minimize the damage caused by positive pressure ventilation. The search for improved ventilatory protocols commenced at the beginning of the 2000s, with ARDS patients in mind in particular, and it was then that the term protective ventilation was first coined. Protective ventilation initially involved a reduction in the tidal volume, an adjustment of the PEEP values to ensure the highest possible pulmonary recruitment, and a plateau pressure limit set at 30 cm H_2_O [56]. Given the clinical relevance of protective ventilation, it was soon extended to the management of mechanical ventilation in the operating room. The daily application of these principles has led to the continuous refinement of lung parenchyma protection protocols—developing the concept of best PEEP, permissive hypercapnia, lung recruitment maneuvers, and, above all, driving pressure [57]. Despite the significant physiopathological rationale, standard protocols for protective ventilation have yet to be identified, mainly due to the ambiguity of the scientific literature [58].

Pressure support ventilation (PSV) is more efficient in limiting ventilator-induced diaphragmatic dysfunction compared with continuous mandatory ventilation (CMV) provided that an adequate level of support pressure and synchronization with the ventilator are maintained [52, 59]. Recently, neurally adjusted ventilator assist (NAVA^®^) has been introduced as a more “physiological” ventilation method that is, at least potentially, able to prevent both overload and disuse atrophy of to the diaphragm allowing early weaning [60]. NAVA delivers assistance in proportion to and in synchrony with the patient’s respiratory efforts, measured as the electrical activity of the diaphragm (EAdi). In this way, it is possible to monitor the force generated by the diaphragm, which is inversely proportional to the amplitude of the electrical stimulus necessary for its contraction [61]. Di Mussi and colleagues, in a recent randomized study on patients in the ICU, compared NAVA with PSV [62]. All the diaphragmatic efficiency indexes, such as the pressure–time produced by the diaphragm, were higher in the group of patients receiving NAVA. Furthermore, episodes of asynchrony were more frequent in the PSV group.

NAVA has the advantage of improving patient–ventilator interaction, synchrony, and diaphragm contractile efficiency [52, 62, 63]; however, the neural component of ventilator-induced diaphragmatic dysfunction remains to be fully understood. Further studies are thus needed to elucidate the mechanisms involved in VIDD with the ultimate aim of developing more effective weaning strategies.

## Conclusion

The ideal time and conditions for achieving successful weaning from mechanical ventilation are a hotly debated topic. As such, weaning remains a non-standardized process and an ever growing proportion of the related literature is focused on trying to identify predictive factors. A complete understanding of the underlying mechanisms responsible for weaning failure is still far from being elucidated. Numerous studies are focused on searching for the individual components involved in respiratory function that may determine weaning failure. Progress has been made though, in particular, thanks to the increasing use of ultrasound that provides real-time in vivo visualization of these components and a form of quantification.

With so many questions about the weaning process remaining open, the purpose of this two-part review was to provide a 360° view of this critical procedure, one that observes the integrated vision of both respiratory function and the most state-of-the-art predictive indexes available to date.

## Data Availability

Not applicable.

## Abbreviations

LUS: Lung ultrasound
IMV: Invasive mechanical ventilation
ARDS: Acute respiratory distress syndrome
PEEP: Positive end-expiratory pressure
ICU: Intensive care unit
SBT: Spontaneous breathing trial
GCS: Glasgow Coma Scale
IAH: Intra-abdominal hypertension
IAP: Intra-abdominal pressure
PLEFF: Pleural effusion
VILI: Ventilator-induced lung injury
VIDD: Ventilator-induced diaphragm disfunction
PSV: Pressure support ventilation
EAdi: Electrical activity of the diaphragm
NAVA: Neurally adjusted ventilator assist
